# Integrated bulk and single-cell transcriptomic profiling reveals bromocriptine sensitivity genes and cellular targets in adenomyosis

**DOI:** 10.12669/pjms.42.2.12639

**Published:** 2026-02

**Authors:** Yuqiang Zhang, Danfen Luo, Zhaomei Zhong, Juan Chen

**Affiliations:** 1 Yuqiang Zhang Gynecology Department, Shenzhen Bao’an Shiyan People’s Hospital, China; 2 Danfen Luo Anesthesiology Department, Shenzhen Long’gan Fifth People’s Hospital, China; 3 Zhaomei Zhong Gynecology Department, Shenzhen Bao’an Shiyan People’s Hospital, China; 4 Juan Chen Gynecology Department, Shenzhen Bao’an Shiyan People’s Hospital, China

**Keywords:** Adenomyosis, Bromocriptine, Differential expression genes, Gynecology, Sensitivity gene, Single cell RNA sequencing

## Abstract

**Objective:**

Bromocriptine has emerged as a potential treatment for adenomyosis. This study aimed to identify genes associated with Bromocriptine sensitivity and explore the biological processes and cell-type-specific expression patterns involved.

**Methodology:**

A cross-sectional bioinformatics study was carried out at Shenzhen Baoan Shiyan People’s Hospital from August 2023 to January 2025. We performed differential expression analysis on endometrial RNA-seq data from patients with adenomyosis before and after Bromocriptine treatment. Functional enrichment analysis was conducted to identify pathways influenced by treatment. Candidate genes were validated in two datasets by comparing normal and adenomyotic endometrial tissues. Single-cell RNA sequencing analysis on eutopic, ectopic and control endometrial samples was conducted to further explore their cellular and molecular regulation pathways.

**Results:**

Differential expression genes after Bromocriptine treatment, with enrichment in pathways such as hormone response, connective tissue development and extracellular matrix organization. Cross-validation with external datasets identified nine candidate genes as Bromocriptine sensitivity markers. Single-cell analysis revealed distinct cellular origins for these genes, with ADAM12 and HOXA11 enriched in smooth muscle and epithelial cells and SFRP1 and SESN3 predominantly expressed in fibroblasts. Notably, several genes showed increased expression in ectopic lesions, supporting their involvement in disease progression and treatment response.

**Conclusion:**

Our integrative analysis identified key genes and pathways associated with Bromocriptine sensitivity in adenomyosis. These findings provide insights into the drug’s mechanism of action and highlight potential biomarkers and therapeutic targets.

## INTRODUCTION

Adenomyosis is a common gynecological disorder characterized by the presence of ectopic endometrial tissue within the myometrium, leading to severe dysmenorrhea, abnormal uterine bleeding, and infertility.^1^,^2^ It is estimated to affect approximately 10% of reproductive-aged women, with prevalence rates reaching 20–35% among patients undergoing hysterectomy.^3^,^4^ Despite this considerable burden, the pathogenesis of adenomyosis remains poorly understood, which complicates the development of effective, mechanism-based therapeutic strategies. Current treatments, including hormonal therapies and surgical interventions, mainly provide symptomatic relief rather than targeting the underlying disease processes.^5^

Bromocriptine is a dopamine agonist primarily used to treat hyperprolactinemia and Parkinson’s disease. Its therapeutic effects are thought to be mediated mainly through inhibition of prolactin secretion. Evidence from endometriosis models suggests it may also inhibit angiogenesis and fibrosis, supporting the hypothesis of a broader mechanism in adenomyosis. Recently, bromocriptine has emerged as a potential therapeutic agent for adenomyosis,^6^ and clinical studies have reported encouraging evidence supporting its efficacy.^7^,^8^ However, the precise molecular mechanisms by which bromocriptine alleviates adenomyosis-related symptoms remain largely unexplored. Elucidating these mechanisms is essential for optimizing its clinical use and identifying biomarkers predictive of treatment response.

The pathophysiology of adenomyosis involves complex molecular and cellular alterations, including dysregulated gene expression, inflammatory responses, and aberrant tissue remodeling. Previous studies have highlighted several genes implicated in the disease, such as MMP2, which is associated with extracellular matrix remodeling, and FSHR, the receptor for follicle-stimulating hormone.^9^,^10^ These gene alterations contribute to the invasive growth and impaired function of ectopic endometrial tissue. An in vitro study has further suggested that bromocriptine may exert therapeutic effects in adenomyosis by modulating signaling pathways related to cell proliferation and apoptosis^11^. Genes that change significantly after treatment may therefore serve as biomarkers of therapeutic response or potential targets for new interventions.

This study aimed to identify genes associated with bromocriptine sensitivity in adenomyosis through comprehensive transcriptomic analysis. Using three datasets from the GEO database, we performed differential expression analysis to identify differentially expressed genes (DEGs) in endometrial tissues from adenomyosis patients before and after bromocriptine treatment. We then conducted functional enrichment analyses to elucidate the biological processes and pathways significantly affected by bromocriptine and constructed a Gene Ontology (GO) enrichment network to map the key pathways involved.

## METHODOLOGY

This was a retrospective bioinformatics study based on publicly available transcriptomic datasets obtained from the GEO and SRA databases. All analyses were performed at Shenzhen Baoan Shiyan People’s Hospital between August 2023 and January 2025, and the study protocol was approved by the institutional ethics committee (2024SL006; dated January 24, 2024). Three bulk RNA-sequencing (RNA-seq) datasets (GSE171653^11^, GSE228005^12^ and GSE78851^13^) were retrieved from the GEO database. The GSE171653 dataset comprises RNA-seq data from endometrial tissues of 11 patients with diffuse adenomyosis, including 11 pre-treatment samples and 10 post-treatment samples after six months of bromocriptine therapy. The GSE228005 dataset provides RNA-seq data from normal and ectopic endometrial tissues of five adenomyosis patients. The GSE78851 dataset includes microarray expression data from normal endometrial tissues of three adenomyosis patients and ectopic endometrial tissues from five controls. In addition, a single-cell RNA-seq (scRNA-seq) dataset containing eutopic endometrium (AM_EM, SRR12791872), ectopic endometrium (AM_EC, SRR12791871) and control endometrium (AM_CTRL, SRR12791873) was obtained from the Sequence Read Archive (SRA).^14^

This study aimed to identify genes associated with bromocriptine sensitivity in adenomyosis by integrating bulk RNA-seq and scRNA-seq analyses. First, differentially expressed genes (DEGs) before and after bromocriptine treatment were identified in the GSE171653 dataset. Functional enrichment analysis was then performed to characterize the biological pathways modulated by treatment. To validate the potential therapeutic relevance of these DEGs, their expression patterns were further examined in normal and ectopic endometrial tissues from the GSE228005 and GSE78851 datasets. Finally, the scRNA-seq dataset was used to assess the cell-type specificity of candidate genes and to compare cell-type composition and gene expression across control, eutopic and ectopic endometrium.

### Identification of adenomyosis DEGs associated with Bromocriptine therapy:

We first applied a pre-filtering step to eliminate genes with low counts, ensuring robust downstream analyses. The filtering retained genes showing a minimum expression level across samples, specifically those with counts of 10 or higher in at least as many samples as the smallest group in our study. Next, we used the DESeq2 package in R (v4.3.1) for differential gene analysis, grouping samples by pre-treatment and post-treatment conditions. We identified candidate differentially expressed genes (DEGs) based on a log2-transformed fold-change (lg2FC) greater than 0.585 (fold change > 1.5 times) and a p-value < 0.05.

### Functional enrichment analyses on Bromocriptine related DEGs:

For the Bromocriptine therapy related DEGs, we used the online platform Metascape (http://metascape.org) to perform enrichment analysis. Metascape performed enrichment analysis based on multiple databases, including KEGG Pathway, GO Biological Processes, Reactome Gene Sets, Canonical Pathways, CORUM, WikiPathways and PANTHER Pathway. The enriched function terms were clustered according to their similarity (kappa score), with function terms having a kappa score > 0.3 being grouped together. Clusters were filtered to retain those with function terms meeting the criteria of p < 0.01, enrichment factor > 1.5 and count > 3. The adjusted p-values were calculated using the Benjamini-Hochberg procedure. The most statistically significant term within each cluster was selected to represent the cluster. This analysis focused on identifying the primary enriched biological processes, cellular components, molecular functions and other biological pathways. We then utilized online biological tools to visualize the results.

### Identification of Bromocriptine sensitive DEGs for adenomyosis:

We further explored the expression differences of these Bromocriptine therapy related DEGs in normal versus adenomyosis-affected tissues using the GSE228005 and the GSE78851 datasets. RNA-seq data of GSE228005 was analyzed using the DEseq2 package and microarray profiling data of GSE78851 was analyzed using the ‘limma’ package. This analysis aimed to identify Bromocriptine-sensitive genes by using the diseased group as a reference, while the expression levels in the normal group were considered the ideal state of treatment. Bromocriptine-sensitive DEGs were defined by the criteria log2FC > 0.585 and false discovery rate (FDR) adjusted *p* < 0.05.

### Single-cell RNA-seq data processing and clustering:

scRNA-seq data from eutopic and ectopic endometrium tissues (AM_CTRL, AM_EM, AM_EC) were processed using Cell Ranger and analyzed with Seurat (v5.0). After quality control filtering (mitochondrial genes >20%, hemoglobin genes >1%, ribosomal genes <1%), normalization and identification of highly variable genes, datasets were integrated using the Harmony algorithm to correct for batch effects. Cells were clustered (resolution = 0.4) and visualized via UMAP. Marker genes were identified using Wilcoxon rank-sum test.

### Cell type annotation and focus gene analysis:

Clusters were annotated manually based on canonical cell-type markers, identifying major populations such as epithelial cells, smooth muscle cells, endothelial cells, fibroblasts, T cells, macrophages, mast cells and inflammatory monocytes. To investigate the cellular origin and expression patterns of the candidate genes, we visualized their distribution using Feature Plot, Dot Plot and violin plots. Gene expression proportions within each cluster and between experimental groups (AM_CTRL, AM_EM, AM_EC) were statistically compared using the Wilcoxon test with FDR correction.

## RESULTS

After data preprocessing and filtering out low-expression genes, a total of 10,347 genes were retained for differential expression analysis. Using the DESeq2 package, we identified 180 genes that met the predefined significance thresholds (log2FC > 0.585, *p* < 0.05) and were differentially expressed before and after Bromocriptine treatment. Of these, 90 genes were upregulated and 90 genes were downregulated after Bromocriptine treatment compared to pre-treatment levels. The DEGs were represented in supplemental Table-SI.

**Table-I:** Differential expression genes of Bromocriptine sensitivity.

Symbol	GSE171653		GSE228005			GSE78851		
	post-therapy vs. prior therapy		normal vs. lesioned tissue			normal vs. lesioned tissue		
	log2FC	p value	log2FC	p value	FDR	log2FC	p value	FDR
ADAM12	1.01	0.0377	3.80	1.23E-10	6.35E-09	3.07	2.58E-07	4.39E-06
CDH11	0.74	0.0402	0.68	0.0029	0.0186	1.83	4.27E-05	0.0002
DIO2	0.84	0.0328	2.35	1.27E-05	0.0002	2.51	9.17E-06	5.20E-05
HOXA11	0.81	0.0368	0.73	6.27E-06	0.0001	2.83	0.0001	0.0003
NT5DC2	0.93	0.0422	0.65	0.0042	0.0240	1.07	0.0023	0.0043
PSD3	0.68	0.0292	0.73	0.0108	0.0480	0.74	0.0023	0.0043
SCUBE2	1.09	0.0021	0.86	0.0070	0.0338	1.26	0.0007	0.0020
SESN3	0.72	0.0043	0.81	0.0086	0.0390	1.71	0.0009	0.0022
SFRP1	0.93	0.0374	1.49	0.0002	0.0018	2.50	3.38E-06	2.87E-05

Dataset and met the significance threshold (FC > 1.5 and FDR < 0.05).

Finally, these 9 genes were identified as Bromocriptine treatment sensitivity genes).

**Table-SI:** Differential expression genes associated with Bromocriptine therapy.

GeneSymbol	Description	log2FC	SE	p-value
AQP3	aquaporin 3 (Gill blood group)	-2.36	0.66	0.0004
SCGB2A1	secretoglobin family 2A member 1	-2.18	0.75	0.0037
NAPSB	napsin B aspartic peptidase, pseudogene	-2.05	0.62	0.0009
PDK4	pyruvate dehydrogenase kinase 4	-1.83	0.70	0.0095
LINC02888	long intergenic non-protein coding RNA 2888	-1.79	0.52	0.0006
HGD	homogentisate 1,2-dioxygenase	-1.78	0.79	0.0237
PTGS1	prostaglandin-endoperoxide synthase 1	-1.62	0.55	0.0034
CFD	complement factor D	-1.54	0.71	0.0302
RXFP1	relaxin family peptide receptor 1	-1.53	0.74	0.0395
IDO1	indoleamine 2,3-dioxygenase 1	-1.50	0.65	0.0207
LGR5	leucine rich repeat containing G protein-coupled receptor 5	-1.49	0.46	0.0013
CFAP300	cilia and flagella associated protein 300	-1.48	0.39	0.0001
SLC3A1	solute carrier family 3 member 1	-1.45	0.62	0.0196
CTNNA2	catenin alpha 2	-1.43	0.62	0.0202
SYT14	synaptotagmin 14	-1.36	0.55	0.0127
TNFSF10	TNF superfamily member 10	-1.35	0.45	0.0028
MGST1	microsomal glutathione S-transferase 1	-1.34	0.51	0.0082
ALDH3B1	aldehyde dehydrogenase 3 family member B1	-1.34	0.51	0.0083
GMPR	guanosine monophosphate reductase	-1.33	0.47	0.0045
TNFRSF10A	TNF receptor superfamily member 10a	-1.33	0.45	0.0032
PRUNE2	prune homolog 2 with BCH domain	-1.31	0.59	0.0265
SLC26A2	solute carrier family 26 member 2	-1.31	0.52	0.0116
LOC112268284		-1.31	0.62	0.0364
PSORS1C3	psoriasis susceptibility 1 candidate 3	-1.28	0.62	0.0372
H2AC6	H2A clustered histone 6	-1.27	0.62	0.0402
PRKX	protein kinase cAMP-dependent X-linked catalytic subunit	-1.26	0.47	0.0073
FBXO6	F-box protein 6	-1.22	0.45	0.0064
RGS2	regulator of G protein signaling 2	-1.20	0.47	0.0106
LPIN3	lipin 3	-1.20	0.58	0.0373
DRC1	dynein regulatory complex subunit 1	-1.19	0.57	0.0383
THEM4	thioesterase superfamily member 4	-1.18	0.53	0.0253
TUBA4A	tubulin alpha 4a	-1.15	0.49	0.0201
SLC34A2	solute carrier family 34 member 2	-1.15	0.41	0.0055
CFAP45	cilia and flagella associated protein 45	-1.14	0.55	0.0368
ANXA2P1	annexin A2 pseudogene 1	-1.14	0.55	0.0398
FTH1P3	ferritin heavy chain 1 pseudogene 3	-1.12	0.44	0.0108
MT2A	metallothionein 2A	-1.11	0.56	0.0473
GBP2	guanylate binding protein 2	-1.11	0.55	0.0438
PPM1H	protein phosphatase, Mg2+/Mn2+ dependent 1H	-1.10	0.49	0.0260
TMEM63A	transmembrane protein 63A	-1.09	0.41	0.0072
MFHAS1	multifunctional ROCO family signaling regulator 1	-1.07	0.49	0.0286
JUNB	JunB proto-oncogene, AP-1 transcription factor subunit	-1.07	0.53	0.0421
GPT2	glutamic--pyruvic transaminase 2	-1.06	0.46	0.0206
TPD52L1	TPD52 like 1	-1.06	0.50	0.0344
RORC	RAR related orphan receptor C	-1.06	0.49	0.0322
ITGB8	integrin subunit beta 8	-1.05	0.47	0.0264
ARG2	arginase 2	-1.05	0.46	0.0216
DAPK1	death associated protein kinase 1	-1.05	0.43	0.0160
ZFP36	ZFP36 ring finger protein	-1.05	0.53	0.0491
AK7	adenylate kinase 7	-1.04	0.44	0.0183
GABARAPL1	GABA type A receptor associated protein like 1	-1.03	0.42	0.0140
BEST1	bestrophin 1	-1.01	0.37	0.0059
FTH1	ferritin heavy chain 1	-0.98	0.36	0.0067
ARL4C	ADP ribosylation factor like GTPase 4C	-0.94	0.47	0.0463
SLC48A1	solute carrier family 48 member 1	-0.94	0.41	0.0225
INO80E	INO80 complex subunit E	-0.93	0.44	0.0357
ST6GAL1	ST6 beta-galactoside alpha-2,6-sialyltransferase 1	-0.92	0.40	0.0225
C4A	complement C4A (Rodgers blood group)	-0.92	0.43	0.0336
C4B	complement C4B (Chido blood group)	-0.92	0.43	0.0316
HOOK1	hook microtubule tethering protein 1	-0.92	0.46	0.0447
TRIB1	tribbles pseudokinase 1	-0.91	0.35	0.0090
LGMN	legumain	-0.90	0.39	0.0206
CMBL	carboxymethylenebutenolidase homolog	-0.90	0.45	0.0468
VTCN1	V-set domain containing T cell activation inhibitor 1	-0.90	0.43	0.0377
JUP	junction plakoglobin	-0.88	0.35	0.0113
BTG2	BTG anti-proliferation factor 2	-0.87	0.37	0.0190
HOOK2	hook microtubule tethering protein 2	-0.86	0.39	0.0268
KCNK1	potassium two pore domain channel subfamily K member 1	-0.86	0.38	0.0235
UCA1	urothelial cancer associated 1	-0.85	0.37	0.0213
ERRFI1	ERBB receptor feedback inhibitor 1	-0.84	0.35	0.0145
STRADB	STE20 related adaptor beta	-0.83	0.39	0.0306
NEDD4L	NEDD4 like E3 ubiquitin protein ligase	-0.83	0.41	0.0435
SECISBP2L	SECIS binding protein 2 like	-0.81	0.41	0.0467
ABHD12	abhydrolase domain containing 12, lysophospholipase	-0.80	0.33	0.0146
PNP	purine nucleoside phosphorylase	-0.80	0.39	0.0423
TSPAN1	tetraspanin 1	-0.79	0.40	0.0484
CNPPD1	cyclin Pas1/PHO80 domain containing 1	-0.78	0.39	0.0439
PLIN2	perilipin 2	-0.75	0.37	0.0439
BCAT1	branched chain amino acid transaminase 1	-0.74	0.32	0.0194
LIMS3	LIM zinc finger domain containing 3	-0.74	0.36	0.0387
ALDH2	aldehyde dehydrogenase 2 family member	-0.74	0.28	0.0088
WARS1	tryptophanyl-tRNA synthetase 1	-0.69	0.31	0.0277
MTMR14	myotubularin related protein 14	-0.67	0.34	0.0481
GPR108	G protein-coupled receptor 108	-0.66	0.32	0.0381
TAFAZZIN	tafazzin, phospholipid-lysophospholipid transacylase	-0.65	0.33	0.0472
LIMS4	LIM zinc finger domain containing 4	-0.65	0.33	0.0486
CCDC97	coiled-coil domain containing 97	-0.65	0.31	0.0379
COX2	cytochrome c oxidase subunit II	-0.61	0.29	0.0374
TALDO1	transaldolase 1	-0.60	0.26	0.0196
ATXN1L	ataxin 1 like	-0.59	0.30	0.0448
FKTN	fukutin	0.61	0.29	0.0345
ANGEL2	angel homolog 2	0.62	0.31	0.0433
EMP1	epithelial membrane protein 1	0.63	0.31	0.0452
SGCE	sarcoglycan epsilon	0.65	0.32	0.0449
USP46	ubiquitin specific peptidase 46	0.66	0.30	0.0248
VCPIP1	valosin containing protein interacting protein 1	0.66	0.27	0.0131
COPS9	COP9 signalosome subunit 9	0.67	0.31	0.0297
PSD3	pleckstrin and Sec7 domain containing 3	0.68	0.31	0.0292
SACS	sacsin molecular chaperone	0.71	0.34	0.0359
PEAK1	pseudopodium enriched atypical kinase 1	0.72	0.36	0.0456
SESN3	sestrin 3	0.72	0.25	0.0043
DPY19L1	dpy-19 like C-mannosyltransferase 1	0.73	0.32	0.0229
MZT2B	mitotic spindle organizing protein 2B	0.73	0.36	0.0420
CDH11	cadherin 11	0.74	0.36	0.0402
CD34	CD34 molecule	0.75	0.37	0.0443
KDR	kinase insert domain receptor	0.76	0.37	0.0412
NR3C1	nuclear receptor subfamily 3 group C member 1	0.76	0.37	0.0412
MEF2C	myocyte enhancer factor 2C	0.76	0.36	0.0369
STIL	STIL centriolar assembly protein	0.76	0.33	0.0209
CAVIN3	caveolae associated protein 3	0.76	0.38	0.0470
PURA	purine rich element binding protein A	0.77	0.38	0.0410
RAB8B	RAB8B, member RAS oncogene family	0.78	0.23	0.0006
SLC12A2	solute carrier family 12 member 2	0.80	0.40	0.0437
LAMA4	laminin subunit alpha 4	0.80	0.37	0.0315
GLT8D2	glycosyltransferase 8 domain containing 2	0.81	0.37	0.0278
HOXA11	homeobox A11	0.81	0.39	0.0368
AFG3L1P	AFG3 like matrix AAA peptidase subunit 1, pseudogene	0.82	0.42	0.0491
GUCY1A2	guanylate cyclase 1 soluble subunit alpha 2	0.83	0.39	0.0345
DIO2	iodothyronine deiodinase 2	0.84	0.39	0.0328
NAP1L3	nucleosome assembly protein 1 like 3	0.84	0.40	0.0353
TBC1D2B	TBC1 domain family member 2B	0.84	0.39	0.0288
CCDC171	coiled-coil domain containing 171	0.85	0.43	0.0496
UNC5C	unc-5 netrin receptor C	0.85	0.43	0.0493
NADK2	NAD kinase 2, mitochondrial	0.86	0.42	0.0397
BAZ1B	bromodomain adjacent to zinc finger domain 1B	0.87	0.41	0.0346
PYGO1	pygopus family PHD finger 1	0.87	0.43	0.0451
ZNF334	zinc finger protein 334	0.87	0.42	0.0363
TIMP1	TIMP metallopeptidase inhibitor 1	0.88	0.44	0.0463
COL21A1	collagen type XXI alpha 1 chain	0.88	0.41	0.0297
RAB30	RAB30, member RAS oncogene family	0.89	0.38	0.0195
TIMP2	TIMP metallopeptidase inhibitor 2	0.89	0.44	0.0437
ARL15	ADP ribosylation factor like GTPase 15	0.89	0.32	0.0047
PABIR2	PABIR family member 2	0.90	0.38	0.0172
FBN1	fibrillin 1	0.91	0.32	0.0042
EMX2	empty spiracles homeobox 2	0.92	0.45	0.0428
NT5DC2	5’-nucleotidase domain containing 2	0.93	0.46	0.0422
SFRP1	secreted frizzled related protein 1	0.93	0.45	0.0374
MXRA8	matrix remodeling associated 8	0.94	0.41	0.0214
GSE1	Gse1 coiled-coil protein	0.95	0.45	0.0366
DST	dystonin	0.95	0.32	0.0031
SLC25A37	solute carrier family 25 member 37	0.95	0.46	0.0409
SH3PXD2B	SH3 and PX domains 2B	0.96	0.39	0.0147
TTC39C	tetratricopeptide repeat domain 39C	0.97	0.48	0.0459
RECK	reversion inducing cysteine rich protein with kazal motifs	0.99	0.48	0.0382
CLEC11A	C-type lectin domain containing 11A	0.99	0.41	0.0166
ECM2	extracellular matrix protein 2	0.99	0.37	0.0066
TUNAR	TCL1 upstream neural differentiation-associated RNA	1.01	0.47	0.0321
PRICKLE2-AS1	PRICKLE2 antisense RNA 1	1.01	0.39	0.0109
ADAM12	ADAM metallopeptidase domain 12	1.01	0.49	0.0377
PTPRD	protein tyrosine phosphatase receptor type D	1.01	0.44	0.0225
ZNF587	zinc finger protein 587	1.02	0.47	0.0306
LTBP1	latent transforming growth factor beta binding protein 1	1.02	0.40	0.0114
MMD	monocyte to macrophage differentiation associated	1.04	0.46	0.0244
HDAC4	histone deacetylase 4	1.05	0.50	0.0360
ZNF264	zinc finger protein 264	1.08	0.51	0.0358
SCUBE2	signal peptide, CUB domain and EGF like domain containing 2	1.09	0.35	0.0021
CAMK4	calcium/calmodulin dependent protein kinase IV	1.09	0.43	0.0118
TMEM120B	transmembrane protein 120B	1.10	0.55	0.0439
PMEPA1	prostate transmembrane protein, androgen induced 1	1.15	0.52	0.0276
KCNIP4	potassium voltage-gated channel interacting protein 4	1.16	0.50	0.0200
MPRIP	myosin phosphatase Rho interacting protein	1.17	0.48	0.0147
TYROBP	transmembrane immune signaling adaptor TYROBP	1.19	0.53	0.0256
BHLHE41	basic helix-loop-helix family member e41	1.23	0.61	0.0428
BGN	biglycan	1.24	0.59	0.0353
ERICH1	glutamate rich 1	1.25	0.56	0.0242
FMN1	formin 1	1.27	0.51	0.0133
STARD10	StAR related lipid transfer domain containing 10	1.28	0.61	0.0358
H1-4	H1.4 linker histone, cluster member	1.30	0.56	0.0197
GABPB2	GA binding protein transcription factor subunit beta 2	1.38	0.52	0.0080
DYNC2I1	dynein 2 intermediate chain 1	1.39	0.58	0.0178
LOC112268323		1.39	0.56	0.0124
LINC01480	long intergenic non-protein coding RNA 1480	1.42	0.43	0.0010
ANK2	ankyrin 2	1.45	0.51	0.0046
COL27A1	collagen type XXVII alpha 1 chain	1.49	0.75	0.0486
IGFBP5	insulin like growth factor binding protein 5	1.55	0.55	0.0050
TNXB	tenascin XB	1.69	0.61	0.0053
SLIT2	slit guidance ligand 2	1.86	0.69	0.0068
MUC5B	mucin 5B, oligomeric mucus/gel-forming	1.93	0.94	0.0392
TUBD1	tubulin delta 1	1.93	0.61	0.0014
CCL21	C-C motif chemokine ligand 21	2.06	0.91	0.0233

### Enriched functional process of Bromocriptine therapy-associated DEGs:

**Fig. 1:**
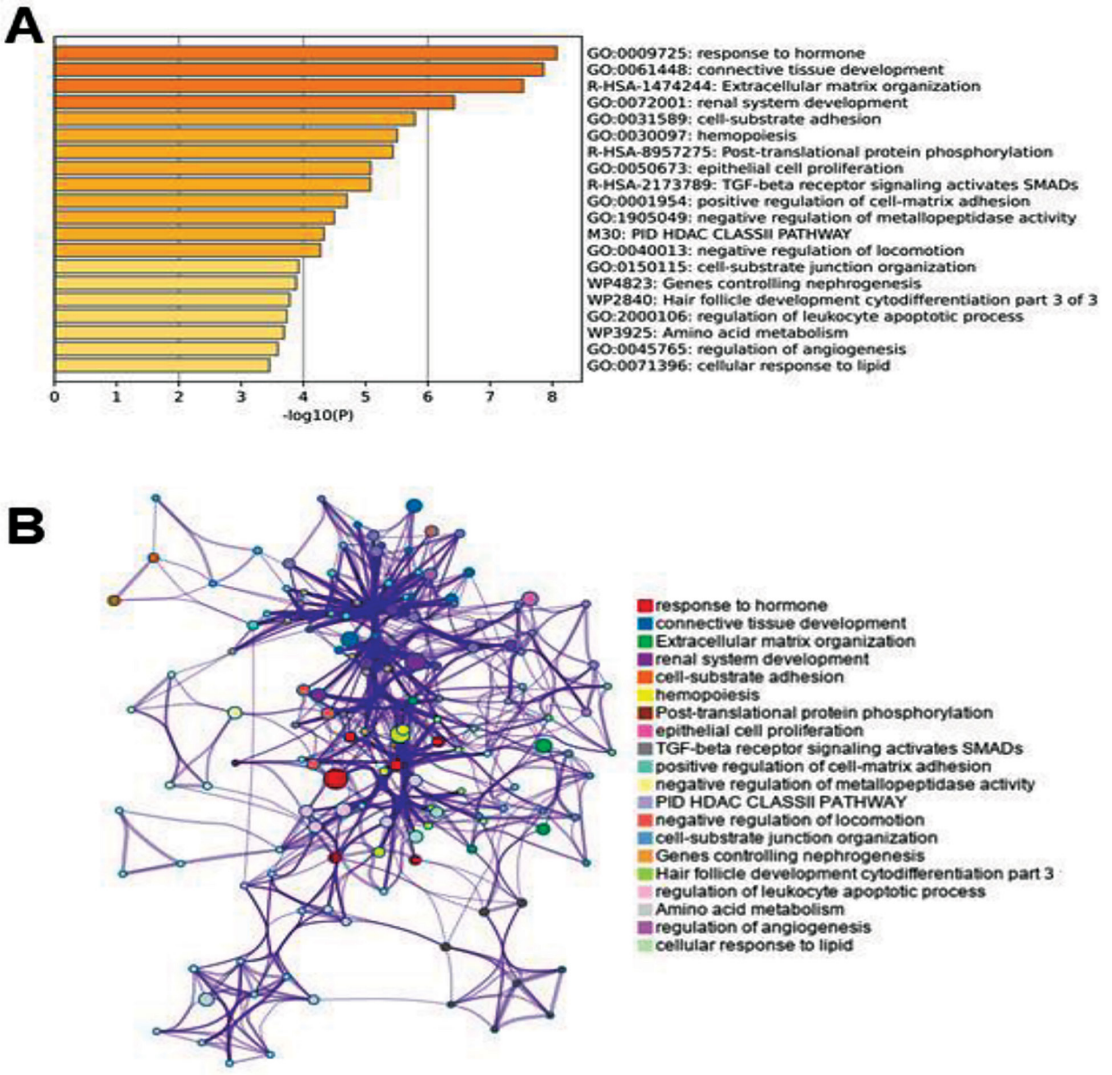
Enrichment analysis on DEGs. (A) Top 20 enriched biological pathways of DEGs associated with Bromocriptine treatment. Bar plot showing the -log10(p-value) of the top 20 functional terms significantly enriched among the DEGs. The most enriched terms include “response to hormone,” “connective tissue development,” and “extracellular matrix organization.” (B) Enrichment network of DEGs before and after Bromocriptine treatment. The network was generated using Metascape based on kappa-statistical similarities among enriched terms (kappa score > 0.3). Nodes represent enriched biological terms, and edges indicate term-term similarity. The network reveals clusters of interconnected terms, highlighting coordinated functional responses, including hormone signaling and tissue remodeling.

The Metascape functional enrichment analysis, based on multiple databases, identified 13 function terms with an adjusted p-value < 0.05. According to statistical significance, the top three terms were response to hormone (GO:0009725), connective tissue development (GO:0061448) and extracellular matrix organization (R-HSA-1474244). Fig.1A displays the top 20 function terms ranked by p-value. To further capture the relationships among the enriched function terms, the enriched network was constructed by the enrichment clusters, where terms with a *Kappa* score > 0.3 were connected by edges (Fig.1B).

### Bromocriptine sensitive DEGs for adenomyosis:

For the DEGs before and after Bromocriptine treatment, we further determined treatment sensitivity genes based on their expression differences in normal versus abnormal endometrial tissues. Out of the 180 candidate genes, 161 were found to overlap in the GSE228005 dataset. Differential expression analysis showed that 35 genes were differentially expressed (log2FC > 0.585, FDR < 0.05) in abnormal endometrial tissues compared to normal endometrial tissues. However, only those genes showing consistent differential expression trends before and after Bromocriptine treatment and also exhibited similar differential expression when comparing normal to abnormal endometrial tissues, were identified as Bromocriptine treatment sensitivity genes. In normal endometrial tissues of the GSE228005, 11 genes were upregulated compared to abnormal endometrial tissues and these genes were also highly expressed in endometrial tissues post Bromocriptine treatment. Additionally, six other genes were downregulated in abnormal endometrial tissues and also exhibited low expression levels before Bromocriptine treatment. These genes were further confirmed using the GSE78851, nine genes upregulated in the normal tissue, showed consistent trend in with the GSE228005 Cell clustering and annotation.

**Fig. 2:**
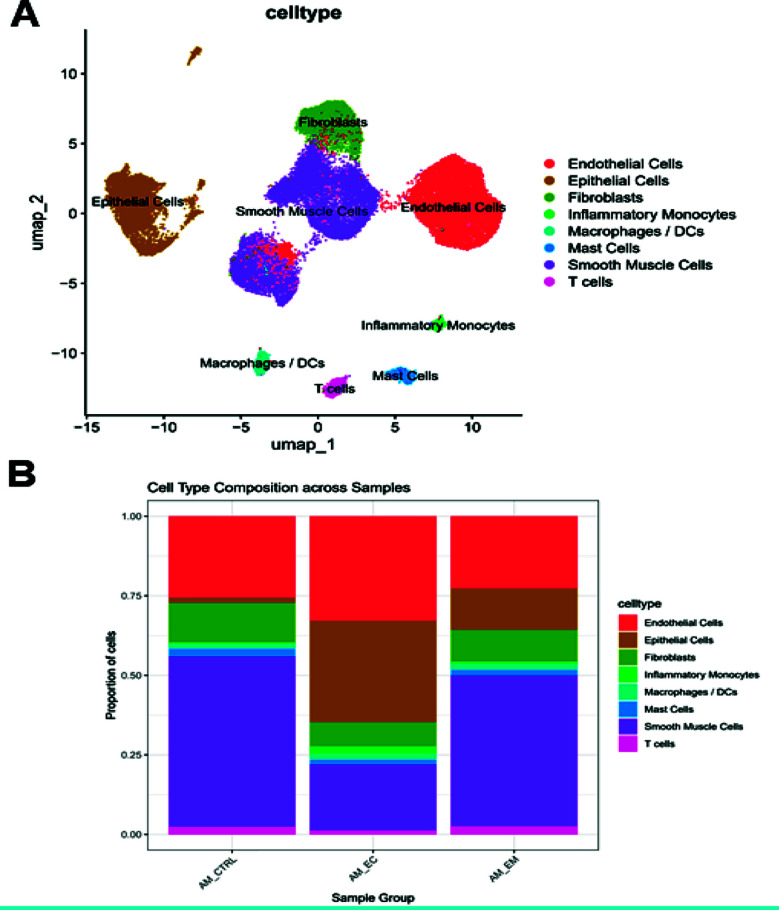
Single cell RNA-sequencing analysis. (A) UMAP visualization of cell type annotations across all samples. Uniform Manifold Approximation and Projection (UMAP) plot showing the distribution of 42,291 single cells across 14 clusters. Cells were annotated into eight major cell types based on canonical markers, including endothelial cells, smooth muscle cells, epithelial cells, fibroblasts, T cells, mast cells, macrophages/DCs, and inflammatory monocytes. Each color represents a distinct cell type. Cells from AM_CTRL, AM_EM, and AM_EC samples are integrated using Harmony batch correction. (B) Barplot showing cell type composition across different sample groups. Stacked bar plot illustrating the proportion of each annotated cell type in the three sample groups: AM_CTRL (control endometrium), AM_EM (eutopic endometrium from adenomyosis), and AM_EC (ectopic endometrium). Notably, epithelial cells and smooth muscle cells were increased in the AM_EC group, suggesting tissue remodeling and invasion in adenomyotic lesions.

After quality control and batch correction, a total of 42,291 cells were retained for downstream analysis. UMAP visualization revealed clear separation of major cell types across samples (Fig.2A). Fourteen clusters were identified and annotated into eight major cell types based on the expression of canonical markers (Table-SII), including endothelial cells, smooth muscle cells, epithelial cells, fibroblasts, T cells, mast cells, macrophages/DCs and inflammatory monocytes. The proportion of each cell type varied between AM_CTRL, AM_EM and AM_EC groups (Fig.2B). Notably, the proportion of Epithelial Cells and Smooth Muscle Cells was markedly increased in the AM_EC group, reflecting potential pathological changes associated with adenomyosis.

**Table-SII:** Marker genes for cell type annotation.

Cluster	Cell Type Annotation	Most Characteristic Marker Genes
0	Endothelial Cells	SELE, VCAM1, ACKR1
1	Smooth Muscle Cells	RGS5, APOE, ABCC9
2	Epithelial Cells	KRT7, MAL2, LAMB3
3	Smooth Muscle Cells	DES, ACTG2, GREM1
4	Endothelial Cells	KDR, CD93, ESM1
5	Fibroblasts	LUM, DCN, APOD, CTSK
6	Smooth Muscle Cells	MYOM2, FOXC2, CDH6
7	Epithelial Cells	PAEP, SCGB2A1, SLC34A2
8	T cells	CD3D, CD3E, CD3G, TRBC1, TRBC2
9	Mast Cells	TPSAB1, TPSB2, CPA3, MS4A2
10	Macrophages / DCs	MRC1, CD36, CCL21
11	Inflammatory Monocytes	IL1B, CCL3, LYZ, CD86
12	Epithelial Cells	KRT6A, KRT6B, S100A8, S100A9
13	Epithelial Cells	S100A7, S100A8, S100A9, CSTA

### Cell-Type-Specific Expression of Focus Genes:

**Fig. 3:**
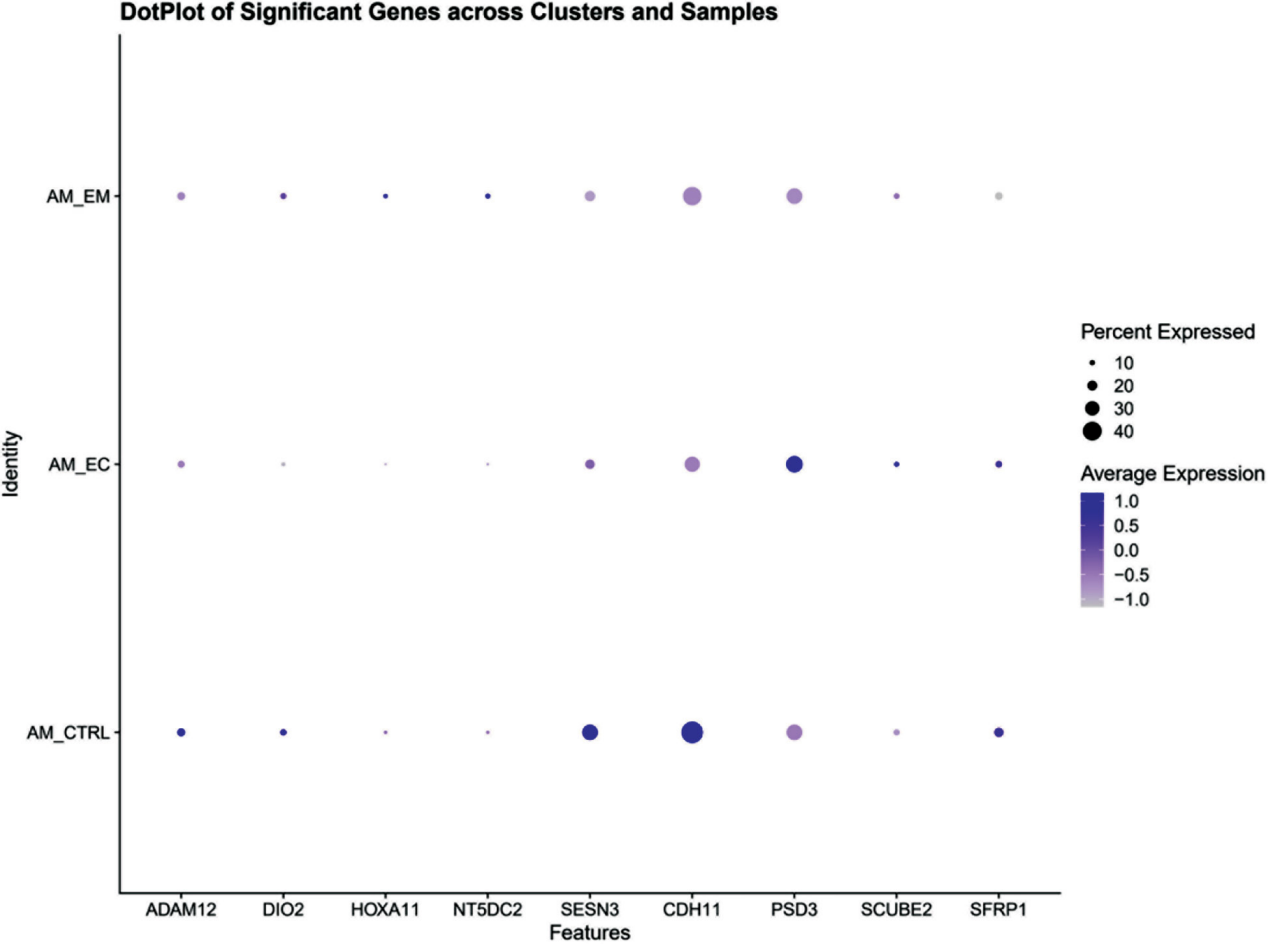
DotPlot of focus genes across cell clusters and groups. (A) DotPlot visualizing the expression and proportion of expressing cells for nine focus genes (e.g., ADAM12, HOXA11, SCUBE2, SFRP1, SESN3) across all 14 clusters. Dot size represents the percentage of cells expressing each gene, while color intensity indicates average expression level. These genes showed distinct cluster-specific expression, highlighting cell-type specificity in Bromocriptine response. (B) DotPlot displaying the expression of the nine candidate genes across different sample groups (AM_CTRL, AM_EM, and AM_EC). Differential expression was evident for multiple genes, such as ADAM12 and HOXA11, particularly in the AM_EC group, indicating their potential role in disease progression and treatment response.

The nine candidate Bromocriptine-sensitive genes displayed distinct expression patterns across clusters. For instance: ADAM12 and DIO2 were specifically enriched in smooth muscle cells, implicating their role in matrix remodeling and muscle regeneration. SFRP1 and SESN3 showed strong expression in fibroblasts, suggesting involvement in Wnt signaling and oxidative stress response. HOXA11, a gene essential for endometrial development, was enriched in epithelial cells and upregulated in AM_EC samples. These results were confirmed by DotPlot visualizations (Fig.3).

### Differential expression of focus genes between groups:

**Fig. 4:**
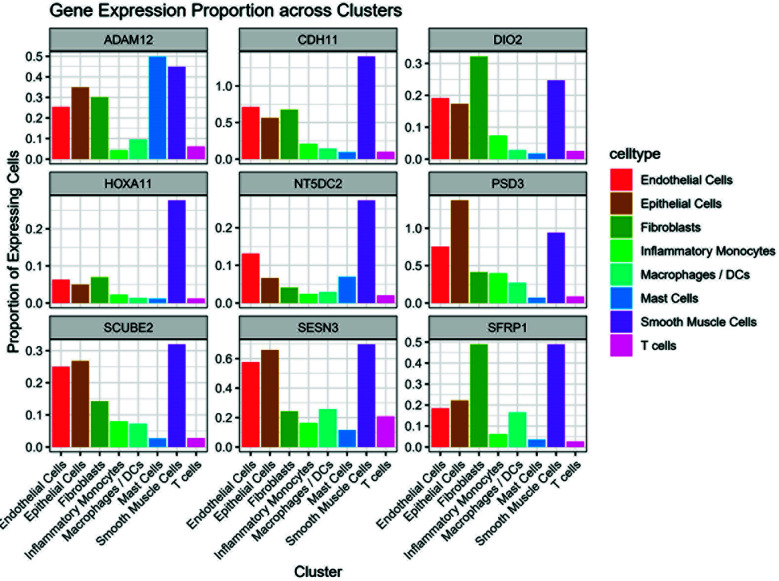
Expression levels of focus genes across cell types and sample groups. Bar plots summarizing the expression proportions of the nine focus genes across annotated cell types within AM_CTRL, AM_EM, and AM_EC groups. Genes such as ADAM12 and HOXA11 showed elevated expression in smooth muscle and epithelial cells in AM_EC tissues, while SFRP1 and SESN3 were enriched in fibroblasts, supporting their role in tissue remodeling and cell-type-specific Bromocriptine sensitivity.

Differential expression analysis revealed that several focus genes showed significant differences between AM_CTRL and AM_EC/AM_EM groups. Particularly, ADAM12 and HOXA11 were significantly upregulated in the AM_EC group compared to controls (FDR < 0.05). These changes were mainly observed in specific cell types, including Smooth Muscle Cells and Epithelial Cells (Fig.4).

## DISCUSSION

Our study is the multi-tiered validation strategy integrating differential expression, independent cohorts, and single cell resolution which robustly identifies cell type specific candidate genes for Bromocriptine response. In this study, we integrated bulk and single-cell transcriptomic data to characterize the molecular response to bromocriptine in adenomyosis. By combining paired endometrial samples before and after bromocriptine treatment with independent datasets of normal and adenomyotic endometrium, we identified nine candidate genes whose expression was consistently dysregulated in disease and shifted toward normal levels following therapy. These bromocriptine-sensitive genes, together with the associated pathways and cell types, provide new insights into the potential mechanisms of bromocriptine action and suggest candidate biomarkers for treatment response.

Pathway enrichment analysis showed that bromocriptine-associated DEGs were significantly enriched in the “response to hormone” pathway. This finding is consistent with previous reports that adenomyosis is characterized by endocrine dysregulation, including disturbances of the hypothalamic pituitary ovarian axis, hyperprolactinemia, and hyperestrogenism.^15^ As a dopamine agonist, bromocriptine primarily inhibits prolactin secretion and may thereby help restore hormonal homeostasis. In addition, membrane progesterone receptors (mPRα, mPRβ) and estrogen receptor β (ERβ) are upregulated in adenomyotic tissues, and estradiol has been shown to markedly enhance the proliferation of adenomyotic cells.^16^ Together, these observations suggest that the heightened hormonal responsiveness of adenomyotic tissue plays a central role in disease pathogenesis and that bromocriptine may exert therapeutic effects, in part, by modulating this axis.

Beyond endocrine regulation, our enrichment results also implicated pathways related to connective tissue development and extracellular matrix (ECM) organization, indicating that bromocriptine may influence tissue remodeling another key pathological feature of adenomyosis. Previous animal studies have demonstrated that bromocriptine ameliorates vascular pathology and metabolic syndrome in rats by modulating connective tissue integrity and fibrosis.^17^,^18^ Consistent with this, several of the bromocriptine-sensitive genes identified in our analysis are functionally linked to ECM dynamics and fibrotic remodeling. For example, ADAM12, which encodes a metalloprotease involved in cell–matrix interactions and muscle regeneration, has been associated with tissue fibrosis and enhanced cell migration.^19^,^20^ CDH11, a cadherin mediating cell–cell adhesion, is associated with multiple fibrotic disorders,^21^ whereas NT5DC2 has been implicated in the regulation of cellular stress and metabolic responses.^22^ These findings support a model in which bromocriptine may modulate ECM composition and fibroblast activity, contributing to structural remodeling of eutopic and ectopic endometrium.

Several other candidate genes point to dysregulation of canonical signaling pathways relevant to cell survival and inflammation. SFRP1, SCUBE2, and SESN3 are known regulators of Wnt,^23^ TGF-β,^24^ and mTOR^25^ signaling, respectively. These pathways control key processes such as proliferation, apoptosis, and inflammatory responses and have been implicated in gynecologic diseases. The observation that expression of these signaling-related genes shifts toward a more normal pattern after bromocriptine treatment suggests that the drug may promote favorable molecular remodeling not only at the level of ECM and connective tissue but also through rebalancing core regulatory pathways in endometrial cells.

To dissect these tissue-level changes at cellular resolution, we leveraged single-cell RNA-sequencing data. Our scRNA-seq analysis revealed substantial heterogeneity in cellular composition across control and adenomyosis samples, with a notable expansion of epithelial and smooth muscle cells in AM_EC tissues, reflecting the pathological hallmarks of invasive lesion growth and tissue remodeling in adenomyosis.^26^ Mapping the nine candidate genes onto specific cell types further refined their potential roles. ADAM12 and HOXA11 were predominantly expressed in smooth muscle and epithelial cells, suggesting that these populations are primary effectors in tissue invasion and fibrotic remodeling. In contrast, SFRP1 and SESN3 were enriched in fibroblasts, supporting their contribution to ECM turnover and fibroblast proliferation.^14^ These cell type–specific expression patterns provide a more nuanced understanding of how bromocriptine may act through distinct cellular compartments within the endometrium.

Moreover, differential expression analyses demonstrated that ADAM12 and HOXA11 were significantly upregulated in the AM_EC group compared with AM_CTRL, particularly within smooth muscle and epithelial compartments, supporting a potential dual role as biomarkers of disease severity and predictors of therapeutic response.^27^,^28^ Collectively, our findings highlight the added value of combining bulk RNA-seq screening with scRNA-seq validation. This integrative approach allows complex tissue-level changes to be decomposed into specific cellular programs and signaling pathways and may inform more precise, cell type–targeted treatment strategies. In particular, therapeutic interventions directed at smooth muscle cells and fibroblast subsets may hold promise as disease-modifying approaches in adenomyosis.^29^

### Limitations:

Nevertheless, several limitations must be acknowledged. First, our analysis was based on publicly available datasets that lacked detailed clinical annotations (e.g., patient age, disease stage, and specific Bromocriptine treatment regimens). These datasets may also harbor inherent batch effects and technical variability, despite our robust normalization efforts. Second, the sample size in the included datasets was modest, limiting statistical power and generalizability. Third, transcriptomic data alone cannot fully capture the complexity of functional outcomes; gene expression changes do not always correlate with protein or metabolite levels.

## CONCLUSION

This study identified nine genes potentially sensitive to Bromocriptine treatment in adenomyosis patients by integrating bulk RNA-seq and single-cell transcriptomic data. These genes are involved in key biological processes such as cell adhesion, ECM remodeling, signaling and stress response, all relevant to adenomyosis pathophysiology. Our findings suggest that Bromocriptine may exert cell type-specific effects by modulating pathways involved in hormonal regulation and tissue remodeling. These insights provide a foundation for developing biomarkers of therapeutic response and guide future precision therapies. However, further functional validation and clinical correlation studies are warranted to confirm their utility in clinical practice.

### Recommendations:

Therefore, future research should integrate proteomic and metabolomic datasets for a more comprehensive view. Finally, while we identified key genes and pathways, functional validation in vitro or in vivo remains essential to confirm their roles and potential as therapeutic targets.
